# A paediatrician's guide to clinical trials units

**DOI:** 10.1136/archdischild-2015-310036

**Published:** 2016-06-10

**Authors:** Chris Gale, Edmund Juszczak

**Affiliations:** 1 Imperial Clinical Trials Unit and Section of Neonatal Medicine, Imperial College London, Chelsea and Westminster Hospital Campus, London, UK; 2 NPEU Clinical Trials Unit, Nuffield Department of Population Health, University of Oxford, Oxford, UK

**Keywords:** Data Collection, Evidence Based Medicine, Health Economics, Information Technology

## Introduction

A clinical trial is a research study that prospectively assigns health-related interventions to people (or groups) typically using randomisation to evaluate the effects on health outcomes. Well-designed, suitably powered, randomised, trials provide the most reliable evidence about the effectiveness (or not) of interventions and should underpin medical practice. Unfortunately, within paediatrics, many common interventions have not been subjected to such rigorous evaluation,^1^ ^2^ leading to variation in both treatments^3^ ^4^ and outcomes (http://www.rightcare.nhs.uk/index.php/atlas/children-and-young-adults/).

Many more high-quality clinical trials are needed before paediatric care is robustly evidence based. Healthcare professionals are ideally placed to inform such trials, but one factor that limits their involvement is the complexity involved: the processes and requirements for trials of investigational medicinal products are illustrated on the clinical trials route map (http://www.ct-toolkit.ac.uk/routemap).

There has been a significant investment in clinical trials research infrastructure over the last decade recognising this complexity. The UK Clinical Research Collaboration Registered Clinical Trials Units (CTU) Network is part of this infrastructure and oversees the registration of CTUs. Registered CTUs need to demonstrate track record, a multidisciplinary team, robust quality assurance systems, statistical input and secure information technology. CTUs exist to help healthcare professionals navigate this complex landscape and assist in developing clinical questions into well-designed studies.

### The history of CTUs

**Figure 1:**
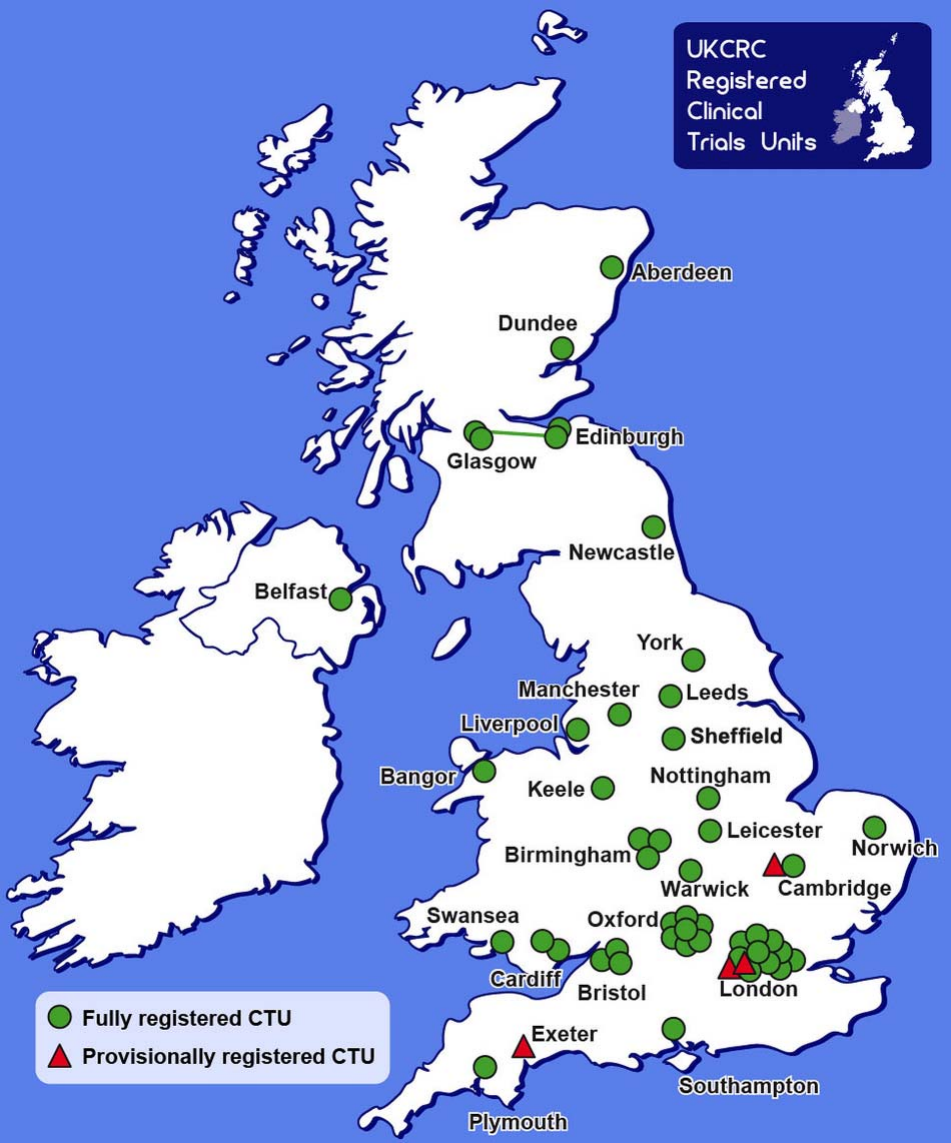
Map of the UK Clinical Research Collaboration (UKCRC), registered clinical trials units (CTUs).

The first UK unit to undertake clinical trials was the Tuberculosis Research Unit in 1948. Further specialist trials units were established in subsequent decades, followed by a more general expansion over the last 20 years. In 2003, a registration process was introduced by the UK Clinical Research Collaboration (UKCRC) to help improve the quality and quantity of UK clinical trials expertise. There are currently 51 registered UK CTUs (http://www.ukcrc-ctu.org.uk/?page=Results) (figure 1), and the National Institute of Health Research (NIHR) provides additional funding to 25 of these to specifically support NIHR projects (http://www.nets.nihr.ac.uk/resources/nihr-ctu).

### The role of a CTU

CTUs have the expertise to provide statistical, epidemiological, logistical and methodological advice, and the coordination to successfully undertake multicentre clinical trials, nationally and internationally. Most CTUs will be able to coordinate clinical trials of investigational medical products in compliance with UK Regulations—specifically, CTUs have the expertise and infrastructure to produce the regulatory compliant documents, databases and monitoring plans required for such trials. CTUs commonly specialise in the trials that they adopt, either by medical specialty (eg, the National Perinatal Epidemiology Unit CTU and perinatal trials) or methodology (eg, the Pragmatic CTU and cluster randomised trials). While there are no dedicated paediatric units, many CTUs support paediatric studies that fall within their area of specialisation. UKCRC CTUs can be searched by disease, methodology or study type at http://www.ukcrc-ctu.org.uk/search/custom.asp?id=468.

**Figure 2:**
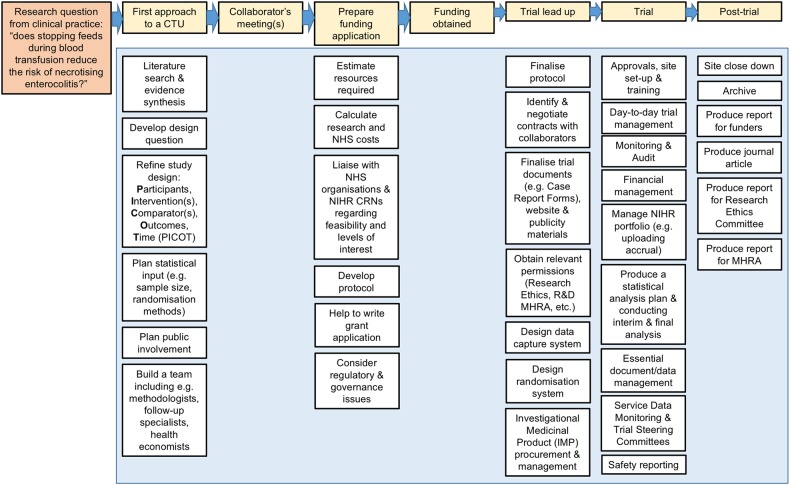
Services and functions of a clinical trials unit (CTU). CRN, Clinical Research Network; MHRA, Medicines and Healthcare Products Regulatory Agency; NHS, National Health Service; NIHR, National Institute of Health Research; R&D, research and development.

CTUs ideally work with a chief investigator from the outset, helping to develop the right research question, setting and methodologically rigorous design. If a trial is successfully funded, the CTU will then usually see it through completion to dissemination. Common services and functions provided by a CTU are summarised in figure 2.

### Working with a CTU

So what are the next steps once you have identified an important research question suitable for a clinical trial? The first step is to formulate the question into Participant, Intervention, Comparator, Outcome format before ensuring that it has not already been answered by checking for recent systematic reviews or performing a literature/systematic review. The NIHR Research Design Service (RDS, http://www.rds.nihr.ac.uk/) can provide assistance where research is likely to fall within remit. A trial is more likely to be funded if it addresses a topic identified as a research priority: priority setting partnerships have been established by the James Lind Alliance (http://www.jla.nihr.ac.uk/) in many specialties like preterm birth.^5^ At this stage, it is important to identify potential funding streams; CTUs can assist with this process, although for clinical studies, funding often entails NIHR streams. Meaningful patient and public involvement is essential for NIHR-funded trials and should be included as early as possible; INVOLVE (http://www.invo.org.uk/) and the RDS can provide guidance here.

Approaching a CTU for support will usually involve an application form or ‘collaboration request’. While it is essential that this process starts at least 3 months before any target funding deadline, the strongest collaborations and proposals often result from an iterative process involving multiple stakeholders including CTUs, public/patient representatives and National Health Service sites, which may often take considerably longer. Any clinical trial needs to eventually (directly or indirectly) lead to tangible patient or health service benefits in order to have a realistic chance of being funded; the application or ‘collaboration’ stage is the CTU's way of determining whether a proposed trial is within remit and is likely to succeed. This is a competitive process, where demonstrating feasibility and eventual patient benefit is key. Any collaboration request will be reviewed within the CTU to judge whether it is competitive and should be supported. If a proposed trial is supported, the resources of the CTU will then work in partnership with the chief investigator to build a strong team to design and deliver the trial, developing the methodological, statistical, organisational and regulatory components in preparation for the next major hurdle, grant submission.

Many funders (including the NIHR) expect clinical trials to be run through a UKCRC registered CTU or require a justification for not doing so; the CTU costs, which incorporate statistical support, trial management, quality control, audit, database management and many other functions, will be included in any grant application. A CTU will also support the complex cost calculations for clinical research using the Attributing the Costs of Health and Social Care Research and Development (AcoRD) framework.

## Summary

Clinical trials are essential to improving paediatric and neonatal care, and paediatricians in tandem with parent and user groups are ideally placed to identify the key questions to be examined in such trials. A CTU can provide expert advice and support in tandem with healthcare professionals to develop a clinical trial; the most fruitful collaborations evolve from early consultation.
